# Clinical and Immunological Features of Human BCL10 Deficiency

**DOI:** 10.3389/fimmu.2021.786572

**Published:** 2021-11-12

**Authors:** Blanca Garcia-Solis, Ana Van Den Rym, Jareb J. Pérez-Caraballo, Abdulwahab Al–Ayoubi, Anas M. Alazami, Lazaro Lorenzo, Carolina Cubillos-Zapata, Eduardo López-Collazo, Antonio Pérez-Martínez, Luis M. Allende, Janet Markle, Miguel Fernández-Arquero, Silvia Sánchez-Ramón, Maria J. Recio, Jean-Laurent Casanova, Reem Mohammed, Rubén Martinez-Barricarte, Rebeca Pérez de Diego

**Affiliations:** ^1^ Laboratory of Immunogenetics of Human Diseases, IdiPAZ Institute for Health Research, La Paz Hospital, Madrid, Spain; ^2^ Innate Immunity Group, IdiPAZ Institute for Health Research, La Paz Hospital, Madrid, Spain; ^3^ Interdepartmental Group of Immunodeficiencies, Madrid, Spain; ^4^ Division of Genetic Medicine, Department of Medicine, Vanderbilt Genetics Institute, Vanderbilt University Medical Center, Nashville, TN, United States; ^5^ Division of Molecular Pathogenesis, Department of Pathology, Microbiology, and Immunology, Vanderbilt Center for Immunobiology, Vanderbilt Institute for Infection, Immunology, and Inflammation, Vanderbilt University Medical Center, Nashville, TN, United States; ^6^ Department of Pediatrics, King Saud Medical City Children’s Hospital, Riyadh, Saudi Arabia; ^7^ Translational Genomics, Centre for Genomic Medicine, King Faisal Specialist Hospital and Research Centre, Riyadh, Saudi Arabia; ^8^ Laboratory of Human Genetics of Infectious Diseases, Necker Branch, Institut National de la Santé et de la Recherche Médicale (INSERM), Paris, France; ^9^ Center for Biomedical Research Network, CIBEres, Madrid, Spain; ^10^ Translational Research in Paediatric Oncology, Haematopoietic Stem Cell Transplantation, Cell Therapy, Instituto de Genética Médica y Molecular (INGEMM)-IdiPAZ, La Paz University Hospital, Madrid, Spain; ^11^ Department of Paediatric Haemato-Oncology and Stem Cell Transplantation, La Paz University Hospital, Madrid, Spain; ^12^ Department of Immunology, 12 de Octubre Hospital, Research Insitute imas12, Complutense University, Madrid, Spain; ^13^ Clinical Immunology Department, San Carlos Clinical Hospital, Madrid, Spain; ^14^ Department of Immunology, Ophthalmology and ENT, School of Medicine, Complutense University, Madrid, Spain; ^15^ St. Giles Laboratory of Human Genetics of Infectious Diseases, Rockefeller Branch, The Rockefeller University, New York, NY, United States; ^16^ Imagine Institute, University Paris Descartes, Paris, France; ^17^ Howard Hughes Medical Institute, New York, NY, United States; ^18^ Department of Pediatrics, Division of Allergy & Immunology King Faisal Specialist Hospital and Research Centre, Riyadh, Saudi Arabia; ^19^ College of Medicine, Alfaisal University, Riyadh, Saudi Arabia

**Keywords:** primary immunodeficiency, combined immunodeficiency, BCL10, autosomal recessive, mass cytometry, computational immunology, CBM complex, next-generation sequencing

## Abstract

The CARD-BCL10-MALT1 (CBM) complex is critical for the proper assembly of human immune responses. The clinical and immunological consequences of deficiencies in some of its components such as CARD9, CARD11, and MALT1 have been elucidated in detail. However, the scarcity of BCL10 deficient patients has prevented gaining detailed knowledge about this genetic disease. Only two patients with BCL10 deficiency have been reported to date. Here we provide an in-depth description of an additional patient with autosomal recessive complete BCL10 deficiency caused by a nonsense mutation that leads to a loss of expression (K63X). Using mass cytometry coupled with unsupervised clustering and machine learning computational methods, we obtained a thorough characterization of the consequences of BCL10 deficiency in different populations of leukocytes. We showed that in addition to the near absence of memory B and T cells previously reported, this patient displays a reduction in NK, γδT, Tregs, and T_FH_ cells. The patient had recurrent respiratory infections since early childhood, and showed a family history of lethal severe infectious diseases. Fortunately, hematopoietic stem-cell transplantation (HSCT) cured her. Overall, this report highlights the importance of early genetic diagnosis for the management of BCL10 deficient patients and HSCT as the recommended treatment to cure this disease.

## Introduction

Primary Immunodeficiencies (PIDs) are a heterogeneous group of diseases that are now referred to as inborn errors of immunity (IEI). The in-depth functional characterization of IEI has been instrumental in improving patient treatment and understanding the mechanisms of human immunity (1). The study of IEI has highlighted the critical and non-redundant roles of the CARD-BCL10-MALT1 (CBM) complex in the proper assembly of human immune response (2). Inborn errors in the components of the CBM complex cause characteristic clinical and immunological consequences (2). Autosomal recessive complete deficiencies in the two Caspase recruitment domain-containing (CARD) adaptor proteins, CARD9 (3) and CARD11 (2, 4) cause isolated invasive fungal infections (3, 5–28) and combined immunodeficiency (CID) respectively (2, 4). Furthermore, bi-allelic loss-of-function (LOF) mutations in the mucosa-associated lymphoid tissue lymphoma-translocation gene 1 (MALT1) causes CID (2, 4). Numerous patients with deficiencies of CARD9, CARD11, and MALT1 have been reported, which has permitted a detailed understanding of the clinical presentation, immunological consequences and best treatment approaches for these three genetic diseases.

IEI in the last member of the CBM complex, B-cell lymphoma/leukemia 10 (BCL10), has been reported in only two unrelated patients to date (29, 30). BCL10 is a 233-amino acid intracellular signaling protein which is part of CBM complex. It is located downstream of various immune receptors and it mediates NF-κB and mitogen-activated protein kinase (MAPK) activation, in a cell type-specific manner (31). The first BCL10 deficient patient was reported in 2014. He was an Amerindian boy who was born to consanguineous parents and died of combined immunodeficiency (CID). The patient had autosomal-recessive complete BCL10 deficiency, resulting in an absence of wild-type (WT) mRNA and protein. The patient experienced multiple infections and active chronic colitis. T-cell and B-cell subpopulations revealed a profound deficit of memory T and B cells, a normal response in myeloid cells to TLR1/2, TLR4, TLR2/6 and Dectin-1 signaling and a strong impairment on nuclear factor kappa-light-chain-enhancer of activated B cell (NF-κB)-mediated fibroblast function (29). The second patient, described in 2020, had autosomal-recessive complete BCL10 deficiency; he is an Asian-Indian boy from India with consanguineous parents. This patient had severe lower respiratory tract infections and immunological studies showed hypogammagloulinemia, without lymphopenia but reduced percentages of memory B cells and memory T cells (30). Hematopoietic stem cell transplantation (HSCT) was the treatment chose for this patient. The present study reports the third unrelated patient with a BCL10 deficiency and analyses the leukocytes of this patient using mass cytometry with unsupervised clustering and machine learning computational methods. The patient is a child from Saudi Arabia with a CID phenotype and a novel *BCL10* mutation resulting in complete autosomal recessive BCL10 deficiency, highlighting the non-redundant role of human BCL10 in immunity.

## Materials And Methods

### Study Approval

The experimental protocol was approved by the ethics committee of La Paz University Hospital (Madrid, Spain), and written informed consent was obtained from the family for participation in this study.

### Human Molecular Genetics and Whole-Exome Sequencing

Genomic DNA was extracted from whole blood with a kit (Qiagen GmbH, Hilden, Germany), according to the manufacturer’s instructions. Whole-exome sequencing (WES) was performed on genomic DNA from whole blood. The libraries were sequenced on an Illumina sequencing platform (mean coverage >80 to 100X).

WES results were validated by polymerase chain reaction (PCR)/Sanger sequencing analysis on genomic DNA from whole blood. PCR was performed with PCR Master Mix (Promega, Fitchburg, WI, USA) and the GeneAmp 9700 PCR System (Applied Biosystems, Foster City, California, USA). The following primer sequences were employed for the genomic coding region of BCL10.

Forward primers (FP): 1F, TCCTCTCCTTCTTCCCCATT; 2F, GCCTGAGCCTCCTGACTTTA; 3F, GATTTGAAATAGATTATGACGGAAA.

Reverse primers (RP): 1R, AGCTCTGCGTTTAGCGATGT; 2R, GGCTGGTCTCAAAACTCCTG; 3R, AAACAAATGATTACAGCCATTTTA.

The PCR products were purified with *ExoSAP-*IT PCR Product Cleanup Reagent (Applied Biosystems) and sequenced with the BigDye Terminator Cycle Sequencing Kit (Applied Biosystems). Sequencing products were purified by precipitating in 70% ethanol, and the sequences were analyzed with an ABI Prism 3700 Genetic Analyser (Applied Biosystems).

### Immunoblots

Human PBMCs were isolated by Ficoll-Hypaque density gradient centrifugation (Amersham-Pharmacia-Biotech, Buckinghamshire, UK) from whole-blood samples obtained from the patient, parents, siblings, and healthy volunteers, and total cell extracts were prepared. Equal amounts of protein from each sample were separated by SDS-PAGE and blotted onto iBlot Gel Transfer Stacks (Invitrogen, Carlsbad, California, USA). These nitrocellulose membranes were then probed with anti-BCL10 rabbit mAb (ab108328, Abcam, Cambridge, MA, USA), followed by a secondary anti-rabbit IgG-HRP linked antibody (Cell Signaling, Beverly, MA, USA). Membranes were stripped and re-probed with an antibody against GADPH (Abcam) as a loading control. Antibody binding was detected by enhanced chemiluminescence (ECL; Amersham-Pharmacia-Biotech).

### Mass Cytometry

#### Staining and Data Acquisition

5x10^6^ peripheral blood mononuclear cells (PBMCs) were stained with Cell-ID Cisplatin-^195^Pt (Fluidigm, South San Francisco, CA) to discriminate dead cells. PBMCs were then FcR blocked with Human TruStain FcX (Biolegend, San Diego, CA), stained with the antibody mix as shown in **Table S2**, fixed with 1.6% paraformaldehyde, and stained with Cell-ID intercalator-Ir. Stained PBMCs were analyzed in a Helios CyTOF 3.0 (Fluidigm) following manufacturer’s instructions at the Cancer and Immunology Core at Vanderbilt University. The data was exported as a Flow Cytometry Standard file (FCS) and normalized using EQ bead standards (Fluidigm) following manufacturer’s protocol.

#### Data Analysis

For multidimensional analysis, the data was pre-gated to remove dead cells, debris, and selection of leukocytes using FlowJo 10.7.2 (Becton, Dickinson & Company, Ashland, OR), as shown in **Figure S1A**. The pre-gated data was exported as an FCS file and then imported into RStudio. We used the package CATALYST (32) to arcsine transform marker intensities with a cofactor of 5 and performed subsequent analysis. Unsupervised clustering was performed using FlowSOM (33), data representation was performed using the R package ggplot2, and marker enrichment modeling (MEM) (34) was used to characterize different clusters. Manual gating was performed in FlowJo, as shown in **Figure S1B**. Frequencies of different populations were exported from FlowJo and analyzed using Prism 9 (GraphPad Software, San Diego, CA).

## Results

### Homozygous *BCL10* Mutation in a Patient With Combined Immunodeficiency

**Figure 1:**
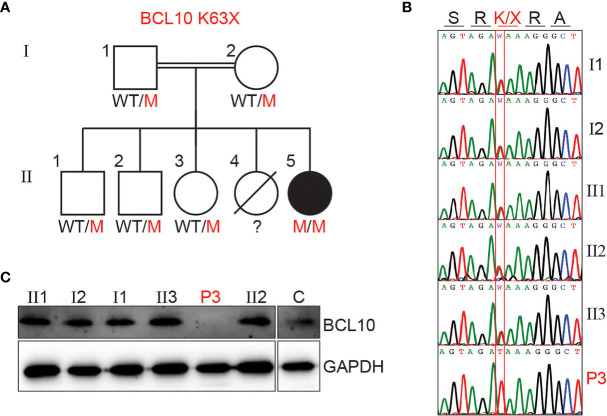
BCL10 deficiency in a patient with CID. **(A)** Familial segregation of the mutation K63X in BCL10. **(B)** Sanger sequencing results of P3 and her family members in the region spanning the BCL10 mutation. The amino acid consequence is indicated above the graphs. **(C)** Immunoblot analysis of BCL10 protein in PBMC of the patient (P3), parents (I1, I2), siblings (II1, II2, II3), and healthy control (C). GAPDH was used as a loading control. The panels illustrate the results from a single experiment, representative of three.

We investigated a female patient of consanguineous parents from Saudi Arabia. She presented at one year of age with fever and bacterial pneumonia that required hospitalization. Her immune workup was suggestive of hypogammaglobulinemia and lymphocytosis (see supplementary material and **Table S1** for detailed clinical history). Furthermore, she had a sister who died at 12 months of age due to a severe chest infection. These observations were compatible with a PID, and she underwent WES. WES revealed a homozygous nonsense mutation (A/T) affecting the nucleotide position g.85270779 (GRCh38.p12) of exon 2 of the gene encoding BCL10 in genomic DNA (gDNA) extracted from leukocytes (g. 85270779A>T). This mutation affects the lysine at position 63 and generates a premature stop codon (c.187A>T, p.K63X). The mutation K63X has not been reported in public databases such as ExAC or gnomAD, suggesting that it is private to this kindred. All other family members tested were healthy and heterozygous for the mutation (**Figures 1A, B**). We then assessed BCL10 expression in PBMC from the patient, healthy controls, and heterozygous carriers. No BCL10 protein was detected in PBMCs from the patient, but BCL10 was detectable in the heterozygous carrier and a healthy donor (**Figure 1C**). Our results indicated that this patient has a BCL10 complete deficiency hence, we will refer to her as P3 since she is the third BCL10 deficient patient described to date (29, 30).

### Overt Immunological Phenotype in BCL10 Deficient Patient

**Figure 2:**
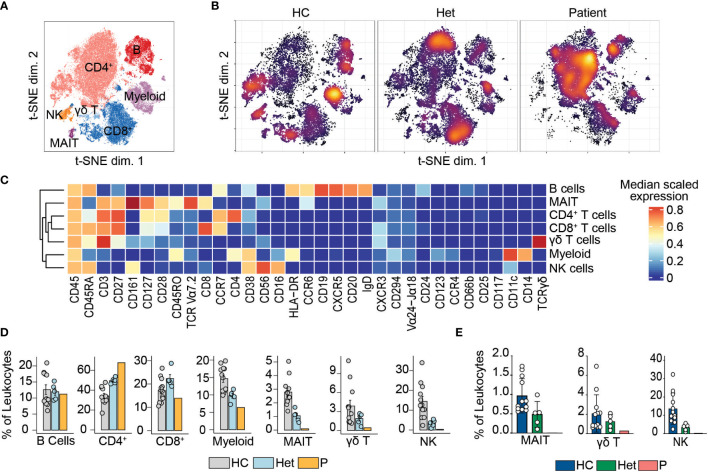
Leukocyte immunophenotyping. **(A)** Dimensional reduction by t-SNE of the 33 markers used for immunophenotyping by mass cytometry. Each color represents a cell population obtained by manual clustering according to their surface marker expression. 50,000 cells from healthy controls (HC), heterozygous carriers (Het), and the patient are represented. **(B)** Density t-SNE showing the distribution of leukocytes in healthy control (HC), heterozygous carrier (Het), and the patient**. (C)** Median expression heatmap of the markers shown under the graph, for the populations shown in **(A, D)** Frequencies of the populations highlighted in **(A)** as a percentage of leukocytes. **(E)** Frequencies of MAIT, γδ T, and natural killer cells in healthy controls (HC), heterozygote carrier (Het), and patient as a percentage of leukocytes obtained by manual gating in FlowJo shown in **Figure S2**.

We previously reported that human BCL10 is critical for the proper development and function of hematopoietic and non-hematopoietic lineages (29). The paucity of patients with BCL10 deficiency and the limited amount of samples obtained from these patients due to the severity of their disease hindered our capacity to survey the consequences of the absence of BCL10 in the overall composition of circulating leukocytes. Furthermore, the low number of individuals reported with deleterious heterozygous variants in BCL10 prevented us from assessing the consequences of BCL10 gene dosage in leukocyte development and function. The samples obtained from P3, her five healthy heterozygous carrier relatives, and the advent of mass cytometry provided the tools to tackle these issues. We performed in-depth immunophenotyping of the patient, healthy heterozygous carriers, and healthy controls using mass cytometry and a cocktail of 33 antibodies directed against surface markers designed to identify most of the common leukocyte populations as well as some rare populations (**Table S2**). Visualization using dimensionality reduction with the t-SNE algorithm revealed marked differences in the distribution of leukocyte populations when comparing a healthy control and heterozygote carrier with the patient (**Figures 2A** **–C**). Of the subpopulations identified by unsupervised clustering followed by manual clustering (**Figures 2A** **–C**), mucosal-associated invariant T cells (MAIT), γδT and natural killer (NK) cells showed a reduction in the patient when compared with healthy controls and heterozygous carriers (**Figure 2D**). This reduction in leukocyte subpopulations is further confirmed by manual gating (**Figures 2E** and **Figure S2**). Considering that the patient was 1.5 years of age at the time of blood draw and the frequencies of MAIT cells at that age range from 0 to 3% of PBMCs, we cannot conclude that the reduction observed in the patient in BCL10 dependent (35). These results suggest that BCL10 deficiency causes changes in the leukocyte population distribution otherwise not seen in healthy individuals. Despite the frequencies of CD4^+^, CD8^+^, B cells, and myeloid cells in the patients falling within normal ranges when compared with healthy controls (**Figure 2D**), the distribution of cells within each of these populations differs in a genotype-dependent manner, suggesting a subtler difference in their subpopulations (**Figures 2A, B**). To further characterize these differences, we reclustered each of these populations and performed an unbiased computational analysis.

### BCL10 Impairs Memory B Cell Differentiation

**Figure 3:**
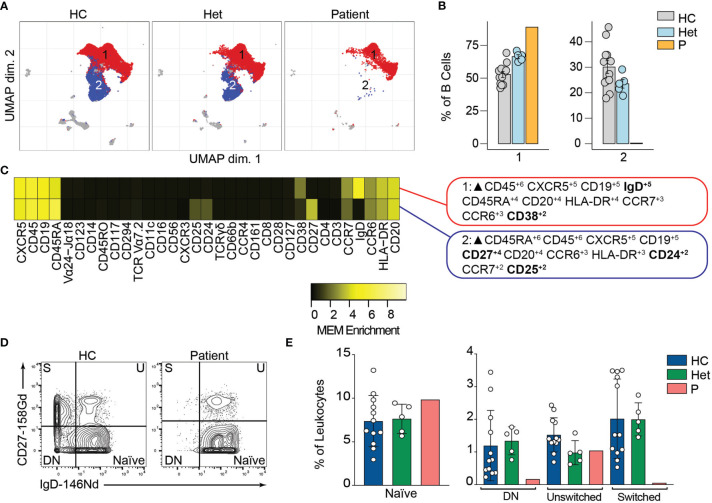
B cells immunophenotyping. **(A)** UMAP representation showing the B cell population from . Each color represents a cluster obtained by unsupervised clustering using flowSOM. 10,000 cells from healthy controls (HC), heterozygous carriers (Het), and the patient are represented. **(B)** Frequencies of the flowSOM clusters highlighted in **(A)** as a percentage of total cells in the B cell population from. **(C)** MEM heatmap and marker tags for the clusters shown in **(A)**. In bold are highlighted the markers differentially expressed between clusters. **(D)** CD27 *vs.* IgD B cell manual gating example for a healthy representative control (HC) and the patient. Detailed gating strategy is shown in **Figure S2**. **(E)** Frequencies of Naïve, Double Negative, switched and unswitched B cells in healthy controls (HC), heterozygote carrier (Het) and patient as a percentage of leukocytes obtained by manual gating in FlowJo.

BCL10 is located downstream of the B-cell receptor (BCR). Upon BCR activation, the LYN kinase phosphorylates the immunoreceptor tyrosine-based activation motifs (ITAMS). These events initiate a molecular cascade that culminates in the activation of PI3K and a phospholipase called PLCγ2. This molecule mediates the formation of diglycerol (DAG) as a second messenger to activate protein kinase C, which will act on CARD11 and promote the formation of the CBM complex. The formation of the CBM complex leads to the activation of the IκB complex kinase (IKK) complex, which will result in the activation of NF-κB (2). Unsupervised clustering in the B cells from **Figure 2** identified two major populations (**Figure 3A**). The frequency of cluster 2 in the patient was severely reduced compared to healthy controls and heterozygous carriers (**Figure 3B**). We used marker enrichment modelling (MEM) to study these two clusters (34). By using machine learning, MEM identifies the markers that distinguish each population allowing for an unbiased characterization of populations. This analysis showed that cluster 1 is characterized by the expression of IgD and CD38 and cluster 2 by CD27, CD24, and CD25 (**Figure 3C**). These markers are consistent with cluster 1 corresponding to naïve B cells and cluster 2 corresponding to memory B cells (36, 37). We confirmed this observation by manual gating and observed that the patient has few detectable double negative or switched memory B cells, but naïve and unswitched compartments comparable to healthy control and heterozygous carriers (**Figures 3D, E**). Our findings and previous reports of BCL10 deficient patients (29, 30) support the critical role of BCL10 in human BCR-dependent signaling and memory B cell generation.

### Reduction of Memory T Cells in the Absence of BCL10

**Figure 4:**
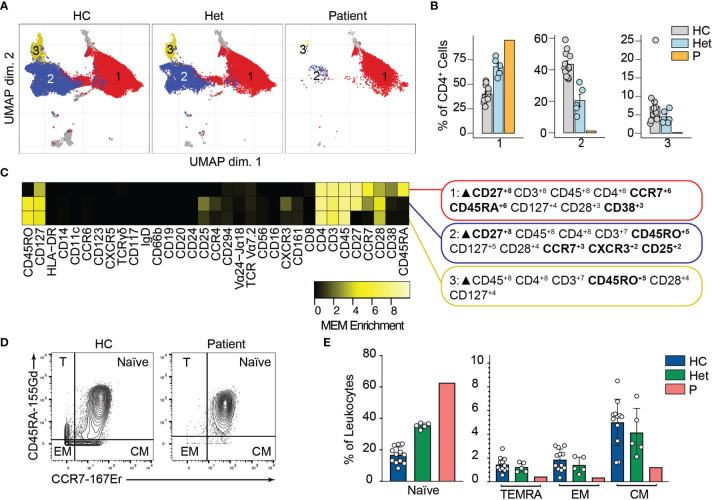
CD4+ T cells immunophenotyping. **(A)** UMAP representation showing the CD4^+^ T population from . Each color represents a cluster obtained by unsupervised clustering using flowSOM. 10,000 cells from healthy controls (HC), heterozygous carriers (Het), and the patient are represented. **(B)** Frequencies of the flowSOM clusters highlighted in **(A)** as a percentage of total cells in the CD4^+^ T cell cluster from . **(C)** MEM heatmap and marker tags for the clusters shown in **(A)**. In bold are highlighted the markers differentially expressed between clusters. **(D)** CD45RA *vs.* CCR7 CD4^+^ T cell gating example for a representative healthy control (HC) and the patient. Detailed gating strategy is shown in **Figure S2**. **(E)** Frequencies of Naïve, TEMRA, effector memory (EM) and central memory (CM) CD4^+^ T cells in healthy controls (HC), heterozygous carrier (Het), and patient as a percentage of leukocytes obtained by manual gating in FlowJo.

We and others have shown that BCL10 is critical for TCR-mediated T cell activation in humans (29). Upon TCR activation, lymphocyte-specific tyrosine kinase phosphorylates ITAMs leading to the activation of PI3K and the phospholipase PLCγ1. As is the case for B cells, these signalling events culminate in the formation of the CBM complex, which will end in the translocation of NF-κB into the nucleus and promote cell proliferation. We studied the consequences of defective TCR-dependent signaling caused by the absence of BCL10 in the composition of the T cell compartment (2). We analyzed CD4^+^ and CD8^+^ T cells (identified in **Figure 2**) separately. By unsupervised clustering of the CD4^+^ cells, we observed three major cell populations (**Figure 4A**). Population 2 and 3 were severely reduced in the patient compared to healthy controls and heterozygous carriers (**Figure 4B**). MEM analysis showed that cluster 1 is characterized by CD27, CCR7, CD45A, and CD38 while cells from cluster 2 express high levels of CD27, CD45RO, CCR7, CXCR3, and CD25, and cluster 3 is characterized by CD45RO expression. This differential marker expression is compatible with cluster 1 corresponding to naïve CD4^+^ T cells and clusters 3 and 4 with memory CD4^+^ T cells (**Figure 4C**). We studied the CD4^+^ memory and naïve compartments by manual gating (**Figures 4D, E**). This analysis showed reduced central memory (CM), effector memory (EM), and TEMRA CD4^+^ T cell compartments with an increased naïve CD4^+^ T cell compartment in the patient when compared to the healthy controls or heterozygous carriers. Furthermore, by manual gating, we also observed a reduction in the frequency of Tregs and T_FH_ in the patient (**Figure S2**). These results suggest that BCL10 is necessary for the differentiation from naïve to memory in CD4^+^ T cells.

**Figure 5:**
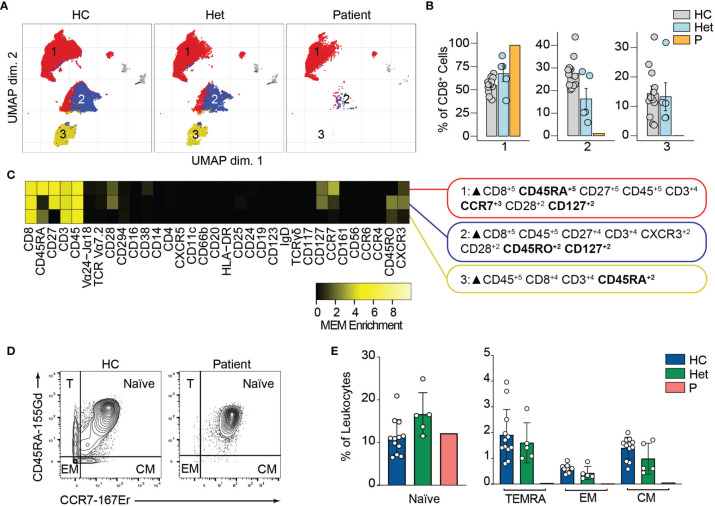
CD8^+^ T cells immunophenotyping. **(A)** UMAP representation showing the CD8^+^ T population from . Each color represents a cluster obtained by unsupervised clustering using flowSOM. 10,000 cells from healthy controls (HC), heterozygous carriers (Het) and the patient are represented. **(B)** Frequencies of the flowSOM clusters highlighted in **(A)** as a percentage of total cells in the CD8^+^ T cells cluster from . **(C)** MEM heatmap and marker tags for the clusters shown in **(A)**. In bold are highlighted the markers differentially expressed between clusters. **(D)** CD45RA *vs.* CCR7 CD8^+^ cell gating example for a representative healthy control (HC) and the patient. Detailed gating strategy is shown in **Figure S2**. **(E)** Frequencies of Naïve, TEMRA, effector memory (EM) and central memory (CM) CD8^+^ T cells in healthy controls (HC), heterozygous carrier (Het), and patient as a percentage of leukocytes obtained by manual gating in FlowJo.

Similarly, unsupervised clustering of the CD8^+^ T cell population rendered three major clusters, two of which were almost absent in the patient compared to the healthy controls and heterozygous carriers (**Figures 5A, B**). Marker characterization by MEM showed that cluster 1 expressed markers characteristic of naïve CD8^+^ T cells such as CD45RA and CCR7. In contrast, clusters 2 and 3 were characterized by the expression of the central memory and effector memory markers (CD45RO, CD127) (38) and TEMRA markers (CD45RA), respectively (**Figure 5C**). We studied the naïve and memory CD8^+^ T cell compartment by manual gating. Similar to our observations in CD4^+^ T cells, we observed an increased naïve CD8^+^ T cell population in the patient compared to the healthy controls or heterozygous carriers. We also observed decreased frequencies of central memory, effector memory, and TEMRA CD8^+^ T cells in P3 (**Figures 5D, E**). The absence of these populations in the patient suggests that BCL10 is necessary for the development of memory CD8^+^ T cells and confirms our previous results (29, 30). As in B cell analysis, we did not observe any difference in the samples from heterozygous carriers compared to healthy controls, suggesting that haploinsufficiency for BCL10 is immunologically silent.

### Normal Myeloid Compartment in BCL10 Deficiency

Mouse studies have shown that BCL10 is critical for NF-κB activation in myeloid cells. This is mediated by the TLR4, TLR2, and TRL6 pathways, which are BCL10 dependent (39–42). Our previous results showed that in humans, BCL10 is redundant for NF-κB activation in these cells (29). We performed unsupervised clustering in the myeloid cluster from **Figure 2**. We studied 10 clusters and observed that the frequencies of these clusters were comparable between healthy controls, heterozygous carriers, and P3 (**Figure S3**). By manual gating, we showed that the frequencies of non-classical monocytes, classical monocytes, intermediate monocytes, myeloid dendritic cells, and plasmacytoid dendritic cells were comparable between healthy controls, heterozygous carriers, and P3 confirming the redundant role of human BCL10 in the development of cells from the myeloid lineage (**Figure S2**).

### Clinical Features in BCL10 Deficiency

P3 had a clinical presentation consistent with the previous two reported BCL10-deficient patients (29, 30) with respect to her bacterial lung infection (supplementary note 1: case report). She had a sister who died at the age of 15 months due to disseminated BCGitis and bacterial sepsis. She did not develop any gastrointestinal manifestations, and her clinical course was cured by HSCT.

## Discussion

In this report, we describe the clinical and immunological consequences of the third human with complete autosomal recessive BCL10 deficiency. We performed cutting-edge immunophenotyping using mass cytometry to understand the effects of BCL10 in the different circulating leukocyte populations. We also applied an unsupervised and machine learning-based computational approach that allowed us to streamline the analysis and interpretation in an unbiased manner. We confirmed the results obtained by this computational approach by performing manual gating. This pipeline can speed up the characterization of the immunological consequences of this and other IEI. Our results showed that BCL10 deficiency impairs the development of memory B, CD4^+^ and CD8^+^ T cells and confirmed previous reports (29, 30). Surprisingly, we observed that unswitched B cells were intact in terms of frequency which, together with the low levels of IgA and IgM in the patients, suggests that the defect observed in B cells is intrinsic to them instead of secondary to the T cell deficiency observed. Furthermore, we observed a reduction in the frequencies of NK, γδT, Tregs, and Tfh cells. Additional experiments are necessary to understand these results better, but our data suggest that BCL10 is critical for the development or function of these cells. Overall, our in-depth immunophenotyping confirmed what we had shown in the previous two patients with BCL10 deficiency and added additional immune cell types affected by the lack of BCL10. Interestingly, when performing in-depth immunophenotyping in the healthy heterozygous carriers we did not observe any detectable defects suggesting that BCL10 haploinsufficiency is immunologically and clinically silent. Our experimental and analytical approach shows the utility of combining mass cytometry with advanced computational methods to better characterize patients with IEI.

Similar to the patient described in this manuscript, both previously reported patients had autosomal recessive complete BCL10 deficiencies leading to a lack of BCL10 protein (29, 30). Our previous work paved the way for the early diagnosis and treatment of P3. The discovery of P3 allowed us to survey the clinical features of human BCL10 deficiency, especially in the early stages of the disease. The first BCL10 deficient patient was reported in 2014. He was an Amerindian boy born of consanguineous parents who died due to severe infections caused by his CID (**Table 1**) (29). He experienced multiple infections since the age of six months, including otitis, encephalitis of unknown etiology, oral candidiasis and diaper dermatitis with *Candida albicans* superinfection, and respiratory viral infections. This patient also had multiple gastrointestinal symptoms, including chronic colitis, prolonged diarrhea due to *Campylobacter jejuni* infection, acute gastroenteritis due to adenovirus, and diarrhea caused by *Clostridium difficile* (29) (**Table 1**). The second patient, an Asian-Indian boy from India with consanguineous parents, was described in 2020 (30) (**Table 1**). This patient had multiple severe lower respiratory tract infections, sometimes requiring hospitalization. At odds with the first case of BCL10 deficiency (29), the second patient was not reported to have significant gastroenteritis and showed a normal gastroesophageal reflux scan (30). HSCT was the option proposed in the patient. The patient presented in this study is the third patient with BCL10 deficiency. As mentioned above, her presentation was consistent with the previous two patients with respect to her bacterial lung infection. She did not develop any gastrointestinal manifestations, and her clinical course was cured by HSCT (**Table 1**).

**Table 1:** Summary of the main genetic, clinical, immunological, and cellular features comparing the three BCL10-deficient patients reported.

	P1	P2	P3
**Mutation**	g.85741978C > T; IVS1+1G>A	g.85270702G>A, c.262C>T, p.R88X	g. 85270779A>T, c.187A>T, p.K63X
**Zygosity**	Homozygosis, consanguineous parents	Homozygosis, consanguineous parents	Homozygosis, consanguineous parents
**Protein expression**	No	No	No
**Main Clinical Features**	6mo: Gastroenteritis, otitis, and respiratory infections. 8mo: Viral infection [flu A and B, adenovirus; respiratory syncytial virus (RSV)]; *acute secondary respiratory failure*, oral candidiasis, and diaper dermatitis. 13mo: Prolonged diarrhea (*Campylobacter jejuni*). 18mo: Diarrhea. Active chronic colitis. 2y 5mo: Acute gastroenteritis (adenovirus) and respiratory infection (RSV). 2y 8mo: Diarrhea (adenovirus). Chronic non-specific colitis. 2y 10mo: Seizures and *status* epilepticus. Secondary *diffuse leukoencephalopathy*. 3y 4mo: Diarrhea (*Clostridium difficile*). D*izziness, disorientation, and generalized weakness with focal abnormal movements.* Suspected encephalitis. Died due to respiratory failure.	1mo: Flare of the BCG scar with increased erythema and swelling. 6mo: Severe viral lower respiratory tract infection and palatal ulcers 8mo and 10mo: lower respiratory tract infections not requiring hospitalization 11mo: Acute onset respiratory distress (*Mycobacterium tuberculosis* was and no evidence fungal infection)	1yo: Hospitalized due to pneumonia (bacterial).
**Cellular phenotype**	B cells: Hypogammaglobulinemia. Lack of memory B cells.	B cells: Hypogammaglobulinemia. Deficit of memory B cells.	B cells: Hypogammaglobulinemia. Deficit of memory B cells.
	T cells: Normal total T cell number. Deficit of memory T cells. T-cell proliferation in response to TCR blocked.	T cells: Normal total T cell number. Deficit of memory T cells.	T cells: Normal total T cell number. Deficit of memory T cells.
	

Keys Suspicion: Clinical history of respiratory infections since the first months of age. No memory lymphocytes (or reduced levels) despite normal total cell numbers, hypogammaglobulinemia. Confirmation: BCL10 sequencing and by measurement of BCL10 protein expression Treatment: HSCT.

Consolidating our knowledge of human BCL10 deficiency, a fast diagnosis and treatment are essential for this kind of patient, as illustrated by the fatal outcome of the first BCL10 deficient patient (29, 43). From the diagnostic standpoint, a clinical history of respiratory infections since the first months of age should raise suspicion for BCL10 deficiency, since this clinical manifestation was present in all three patients reported to date (**Table 1**). Furthermore, a family history of severe respiratory infection in early childhood may indicate this (or other) inherited IEI. All reported BCL10-deficient patients had a sibling who died in the first months of life due to respiratory infections (**Figure 1A**) (29, 43). Additional evidence to suspect BCL10 deficiency may include the absence or severe reduction of memory B and memory T cells and reduced levels of circulating immunoglobulins, comparable to those of patients with hypogammaglobulinemia, as this phenotype has been observed in all 3 reported cases of BCL10 deficiency (29, 30). Finally, BCL10 deficiency should be confirmed by sequencing and functionally testing the putative disease-causing alleles. If BCL10 deficiency is confirmed, HSCT is highly recommended as the treatment of choice, as in CARD11 and MALT1 deficiency. The European Bone Marrow Transplantation and European Society for immunodeficiencies (EBMT/ESID) guidelines for hematopoietic stem cell transplantation (HSCT) for primary immunodeficiencies recommended reduced intensity conditioning. Data from CARD11-BCL10-MALT11 deficiency patients who received a HSCT are scarce (**Table S3**). The conditioning regimen has not been well established and should be adapted considering donor/recipient human leucocyte antigen (HLA) compatibility and patient morbidities. However, reduced intensity conditioning (RIC) based on fludarabine and/or toxicity reduced conditioning using treosulphan followed by HSCT can restore immunological function. Mixed chimerism posttrasplant could be enough to cure and restore clinical phenotype of affected patients. Our experience supports these guidelines since the early diagnosis of P3 allowed for the application of appropriate treatment.

## Data Availability Statement 

The datasets presented in this article are not readily available because the patient withdrew their consent. Requests to access the datasets should be directed to Dr Reem Mohammed.

## Ethics Statement 

The studies involving human participants were reviewed and approved by the ethics committee of La Paz University Hospital (Madrid, Spain). Written informed consent to participate in this study was provided by the participants’ legal guardian/next of kin.

## Author Contributions

BG, AR, and JP-C: Experiments, analysis results, editing. AA-A: Immune cells phenotype, clinical study. LL: Handling of cells, DNA extraction and PBMC processing. CC-Z: Manuscript comments, advice and editing. EL-C: Group leader, consulting on experimental procedures. JM: CyTOF experiments. MF-A, SS-R, and MR: Manuscript comments, advice and editing. J-LC: Manuscript drafting and editing. RM: Physician in charge of the patient’s care. Clinical study. RMB: CyTOF, manuscript drafting and editing. RP: Laboratory head, experiment design, manuscript drafting and editing, corresponding author. AP-M has developed the data about HSCT, LA has helped in response to reviewers and AMA in genetics. All authors contributed to the article and approved the submitted version.

## Funding

Support was provided by FIS grant Ref. PI17/00543, BG is supported by PEJD2019-PRE/BMD-16556 Predoctoral Fellowships CAM. AR was provided support by FIS grant Ref. PI17/00543. JP-C was funded in part by an NIH training fellowship, T32GM139800, Initiative for Maximizing Student Development at Vanderbilt. RMB was funded in part by the CTSA award No. UL1 TR002243 from the National Center for Advancing Translational Sciences.

## Conflict of Interest

The authors declare that the research was conducted in the absence of any commercial or financial relationships that could be construed as a potential conflict of interest.

## Publisher’s Note

All claims expressed in this article are solely those of the authors and do not necessarily represent those of their affiliated organizations, or those of the publisher, the editors and the reviewers. Any product that may be evaluated in this article, or claim that may be made by its manufacturer, is not guaranteed or endorsed by the publisher.
