# Supplementary material for: Clinical and Immunological Features of Human BCL10 Deficiency
Source: Front Immunol. 2021 Nov 12;12:786572. doi: 10.3389/fimmu.2021.786572 (PMC8633570; doi:10.3389/fimmu.2021.786572)
Supplement: Supplementary file 1 [file DataSheet_1.doc]

**This PDF file includes**

**Supplemental data Text note 1**

**Figure S1**

**Figure S2**

**Figure S3**

**Table S1**

**Table S2**

**Table S3**

**References**

**Supplementary note 1: case report**

P3 is a 20 mo girl who presented to the Immunology clinic for newborn screening for a history of a sister with combined immune deficiency, who died at age 15 mo of disseminated BCGitis and bacterial sepsis.

She was closely followed as outpatient and received her killed vaccines. At age 1 year she was admitted to her local hospital with fever and bacterial pneumonia for which she received IV antibiotics. She was treated for oral thrush. Her immune work up showed hypogammaglobulinemia and was started on IVIG and prophylactic bactrim. She was found to have von willebrand disease.

Her WES testing revealed a homozygous mutation in BCL10 gene. She was worked up for hematopoietoic stem cell transplant (HSCT) and received a matched sibling HSCT at age 16 mo. She is alive and followed in our clinic in a stable condition.

**Figures**

**Figure S1. Manual Gating Strategy**


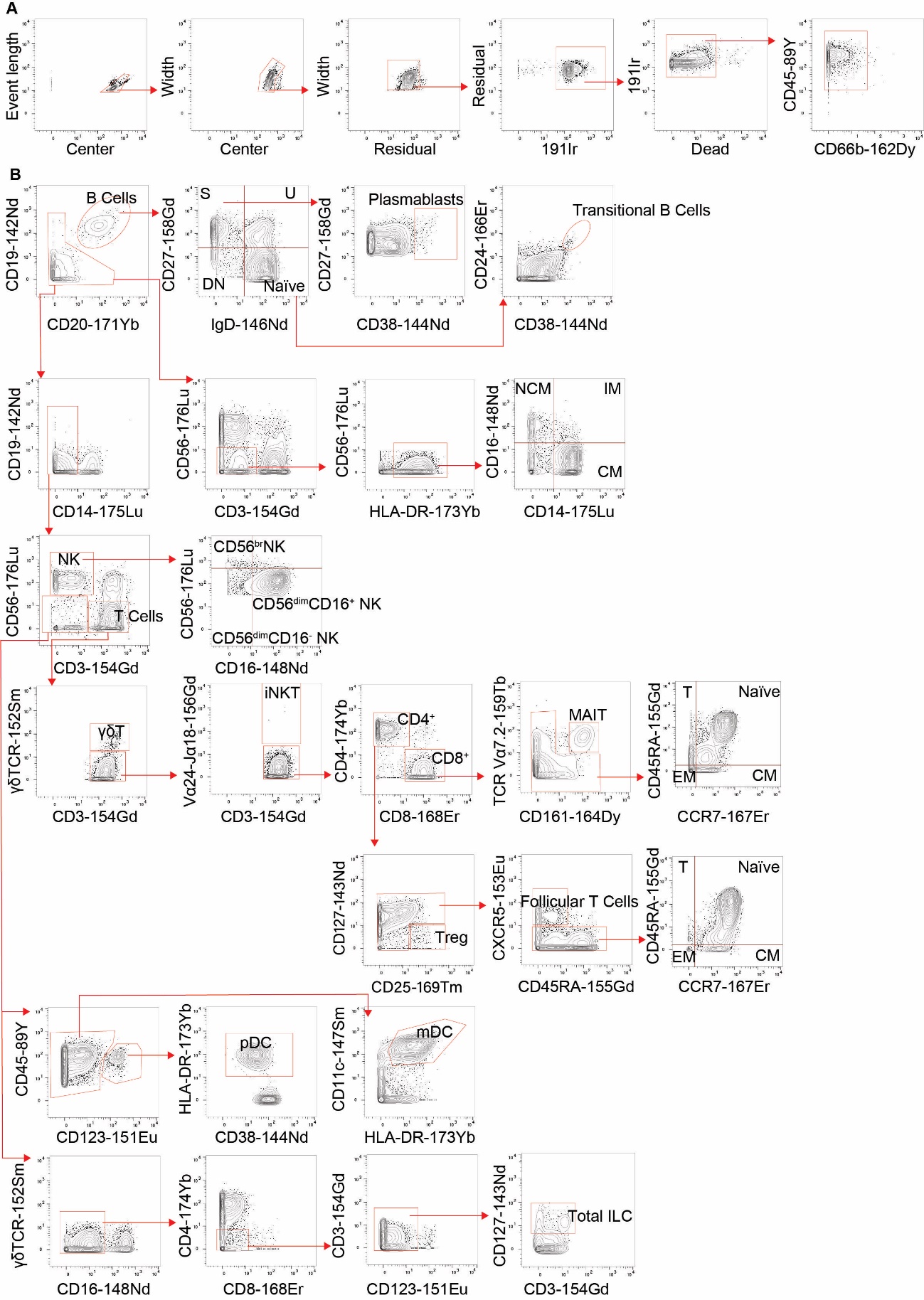


**Figure S1. Manual Gating Strategy**

**(A)** Pre-gating strategy used with mass cytometry data prior the analysis in R. **(B)** Manual gating strategy continuing from (A) used to identify the different immune cell populations described in this manuscript. S correspond to switched, U to unswitched, DN to double negative, NCM to non-classical monocytes, IM to intermediate monocytes, CM to classical monocytes, EM stands for effector memory, CM for central memory and T for TEMRA.

**Figure S2. Immunophenotyping frequencies by manual gating.**

**
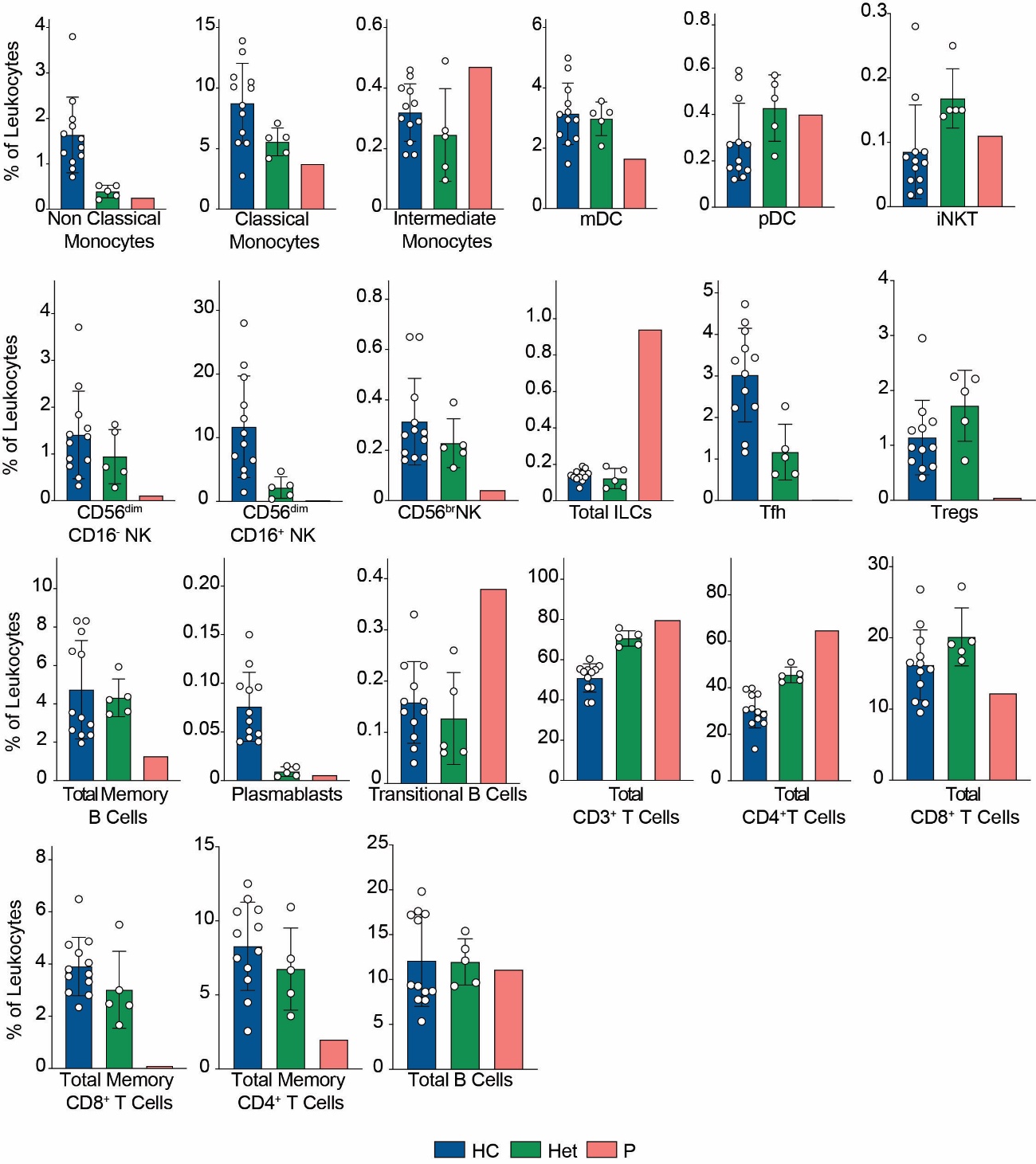
**

**Figure S2. Immunophenotyping frequencies by manual gating.**

Frequencies of different populations as percentage of total leukocytes in healthy controls (HC), heterozygous carrier (Het) and patient following the gating strategy shown in supplementary figure 2.

**Figure S3 Myeloid compartment unsupervised analysis**

**
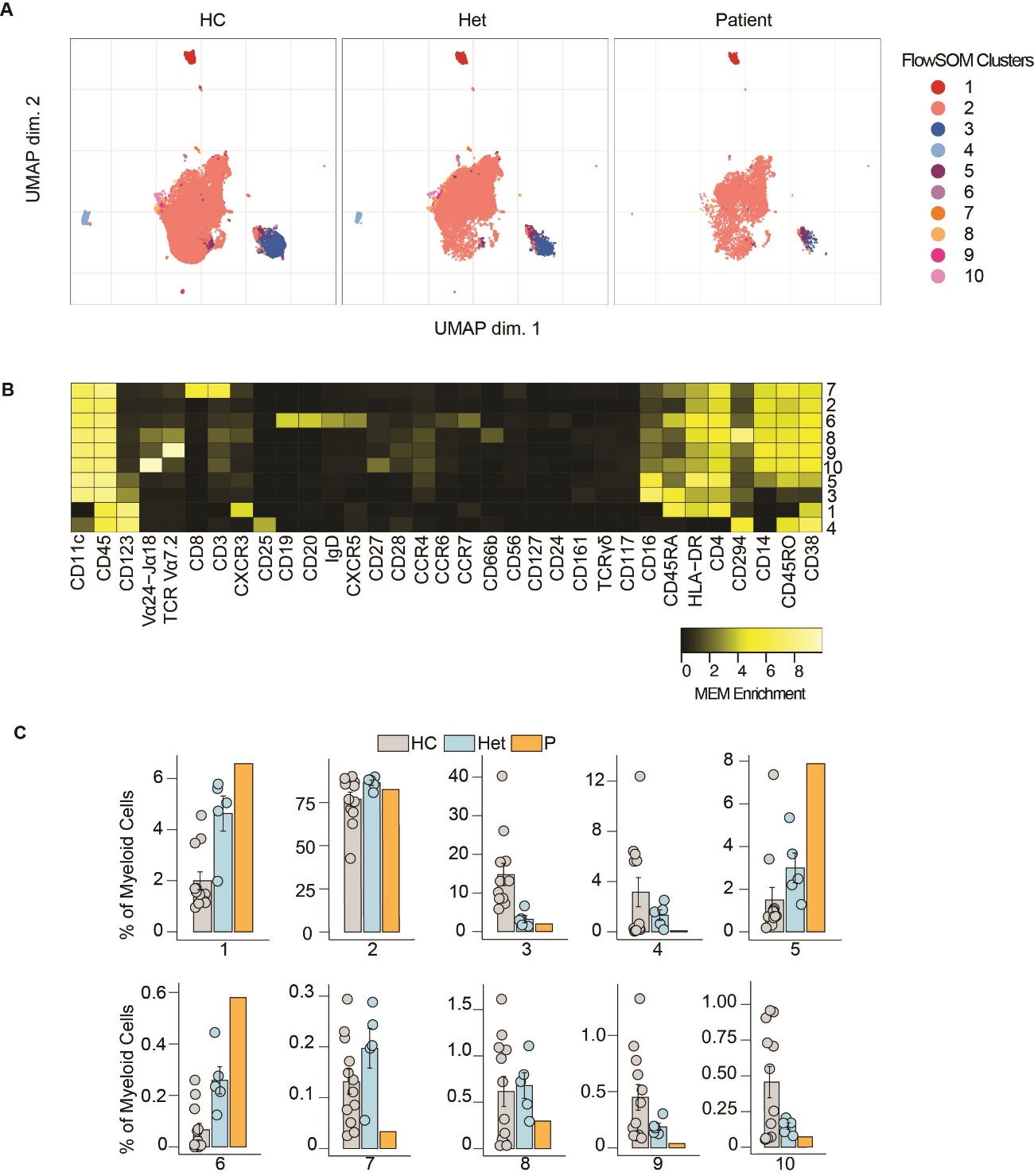
**

**Figure S3 Myeloid compartment unsupervised analysis**

**(A)** UMAP representation showing the myeloid population from figure 2. Each color represents a cluster obtained by unsupervised clustering using flowSOM. 10,000 cells from healthy controls (HC), heterozygous carriers (Het) and the patient are represented. **(B)** MEM heatmap and marker tags for the clusters shown in (A). **(C)** Frequencies of the flowSOM clusters in (A) as percentage of total cells in the myeloid cell population.

**TABLES**

**Table S1. Immune cells populations in peripheral blood and Immunoglobulin (Ig) levels.**

| **A** | **P3** | **Healthy controls^A^** | **B** | | |
| --- | --- | --- | --- | --- | --- |
| **Haemoglobin** | 111 g/L | 115-135 g/L | **Immunoglobulin** | **Values g/L** | **Reference values** |
| **WBC** | 18.99 | 6.000-17.500 | **IgG** | 6.00 | 3.5 – 16.2 g/L |
| **Neutrophils** | 3.98 | 1.500-8.500 | **IgA** | <0.50 | 0.01 – 0.91 g/L |
| **Lymphocytes** | 12.15 | 4.000-10.500 | **IgM** | <0.25 | 0.30 – 1.83 g/L |
| **Monocytes** | 0.9 | 0.200-1.200 |  | | |

**Table S1. Immune cells populations in peripheral blood and Immunoglobulin (Ig) levels**

**(A)** Distribution of P3’s immune cell populations in peripheral blood. Absolute counts x10^9^ per liter of blood. **(B)** Immunoglobulin **(**Ig) levels (IgG, IgA and IgM) measured by nephelometry for P3. ^A^Internal reference values age matched.

**Table S2**. Antibodies used for mass cytometry

| **Antibody** | **Clone** | **Company** | **Isotope** | **Dilution** |
| --- | --- | --- | --- | --- |
| Anti-Human CD45 | H130 | Fluidigm | 89Y | 1:50 |
| Anti-Human CD19 | HIB19 | Fluidigm | 142Nd | 1:50 |
| Anti-Human 127/IL7Ra | A019D5 | Fluidigm | 143Nd | 1:50 |
| Anti-Human CD38 | HIT2 | Fluidigm | 144Nd | 1:50 |
| Anti-Human IgD | IA6-2 | Fluidigm | 146Nd | 1:50 |
| Anti-Human CD11c | Bu15 | Fluidigm | 147Sm | 1:50 |
| Anti-Human CD16 | 3G8 | Fluidigm | 148Nd | 1:50 |
| Anti-Human CD194/CCR4 | L291H4 | Fluidigm | 149Sm | 1:50 |
| Anti-Human CD123/IL-3R | 6H6 | Fluidigm | 151Eu | 1:50 |
| Anti-Human TCRgd | 11F2 | Fluidigm | 152Sm | 1:50 |
| Anti-Human CD185/CXCR5 | RF8B2 | Fluidigm | 153Eu | 1:50 |
| Anti-Human CD3 | UCHT1 | Fluidigm | 154Sm | 1:50 |
| Anti-Human CD45RA | HI100 | Fluidigm | 155Gd | 1:50 |
| Anti-Human CD27 | L128 | Fluidigm | 158Gd | 1:50 |
| Anti-Human CD28 | CD28.2 | Fluidigm | 160Gd | 1:50 |
| Anti-Human CD66b | 80H3 | Fluidigm | 162Dy | 1:50 |
| Anti-Human CD183/CXCR3 | G025H7 | Fluidigm | 163Dy | 1:50 |
| Anti-Human CD161 | HP-3G10 | Fluidigm | 164Dy | 1:50 |
| Anti-Human CD45RO | UCHL1 | Fluidigm | 165Ho | 1:50 |
| Anti-Human CD24 | ML5 | Fluidigm | 166Er | 1:50 |
| Anti-Human CD197/CCR7 | G043H7 | Fluidigm | 167Er | 1:50 |
| Anti-Human CD8 | SK1 | Fluidigm | 168Er | 1:50 |
| Anti-Human CD25 | 2A3 | Fluidigm | 169Tm | 1:50 |
| Anti-Human CD20 | 2H7 | Fluidigm | 171Yb | 1:50 |
| Anti-Human HLA-DR | L243 | Fluidigm | 173Yb | 1:50 |
| Anti-Human CD4 | SK3 | Fluidigm | 174Yb | 1:50 |
| Anti-Human CD56 | NCAM16.2 | Fluidigm | 176Yb | 1:50 |
| Anti-Human CD196 | G034E3 | Fluidigm | 141Pr | 1:25 |
| Anti-Human CD14 | M5E2 | Fluidigm | 175Lu | 1:25 |
| Anti-Human CD117 | 104D2 | Biolegend | 150Nd | 1:25 |
| Anti-Human TCR Vα24/Jα18 | 6B11 | Biolegend | 156Gb | 1:25 |
| Anti-Human TCR Vα7.2 | 3C10 | Biolegend | 159Tb | 1:25 |
| Anti-Human CD294 | BM16 | Biolegend | 161Dy | 1:25 |

**Table S3. Guidelines for hematopoietic stem cell transplantation (HSCT) for CARD11-BCL10-MALT11 deficiency patients.**

|  | **Conditioning** | **Donor** | **Source** | **Graft manipulation** | **Chimerism** | **Outcome** | **Status** | **Reference** |
| --- | --- | --- | --- | --- | --- | --- | --- | --- |
| CARD11 deficiency | Treo/Flu, Thio/Ritux, ATG | Haplo | PB | TCRαβ | Complete | Infecions, GvHD | Died of TRM | (1) |
| CARD11 deficiency | Flu/Bu/Alem | MMUD 9/10 | BM | No | Complete | Infecions, GvHD | Alive with many morbidities | (2) |
| MALT1 deficiency | Cy/Mel/ATG | MMUD 9/10 | PB | CD34^+^ and T cells addback | Mixed | CMV disease | Alive | (3) |
| MALT1 deficiency | Flu/Mel/Alem | MRD | BM | No | Complete | Infections | Alive | (4) |
| MALT1 deficiency | Flu/Bu/Alem | MUD | PB | CD34^+^ | Complete | CMV reactivation | Alive | (5) |
| MALT1 deficiency | Flu/Bu/Alem | MUD | PB | CD34^+^ | Mixed | CMV reactivation | Alive | (5) |
| CARD11 deficiency | Cy/Mel/Alem | MUD | PB | Not reported | Mixed | Not reported | Alive | (6) |
| CARD11 deficiency | Not reported | MRD | Not reported | Not reported | Complete | Not reported | Alive | (7) |

* Treo: Treosulphan; Flu: fludarabine, Thio: thiotepa; Bu: busulfan; Cy: cyclophosphamide; Mel: melphalan; ATG: rabbit anti-thymocyte globulin; Alem: alentuzumab; Ritux: rituximab; PB: peripheral blood; BM: bone marrow; TCRαβ: depletion of TCRαβ T cells; CD34^+^: CD34^+^ cells selection graft; Haplo: haploidentical donor; MMUD: mismatch unrelated donor; MRD: match related donor; TRM: trasplant related mortality.

**References**

1. Lu Y, Li X, Liu S, Zhang Y, Zhang D. Toll-like receptors and inflammatory bowel disease. *Front Immunol* (2018) **9**: doi:10.3389/fimmu.2018.00072

2. Lu HY, Sharma M, Sharma AA, Lacson A, Szpurko A, Luider J, Dharmani-Khan P, Shameli A, Bell PA, Guilcher GMT, et al. Mechanistic understanding of the combined immunodeficiency in complete human CARD11 deficiency. *J Allergy Clin Immunol* (2021) doi:10.1016/j.jaci.2021.04.006

3. Punwani D, Wang H, Chan AY, Cowan MJ, Mallott J, Sunderam U, Mollenauer M, Srinivasan R, Brenner SE, Mulder A, et al. Combined Immunodeficiency Due to MALT1 Mutations, Treated by Hematopoietic Cell Transplantation. *J Clin Immunol* (2015) **35**:135–146. doi:10.1007/s10875-014-0125-1

4. McKinnon ML, Rozmus J, Fung SY, Hirschfeld AF, Del Bel KL, Thomas L, Marr N, Martin SD, Marwaha AK, Priatel JJ, et al. Combined immunodeficiency associated with homozygous MALT1 mutations. *J Allergy Clin Immunol* (2014) **133**:1458–1462. doi:10.1016/j.jaci.2013.10.045

5. Charbit-Henrion F, Jeverica AK, Bègue B, Markelj G, Parlato M, Avcin SL, Callebaut I, Bras M, Parisot M, Jazbec J, et al. Deficiency in mucosa-Associated lymphoid tissue lymphoma translocation 1: A novel cause of IPEX-like syndrome. *J Pediatr Gastroenterol Nutr* (2017) **64**:378–384. doi:10.1097/MPG.0000000000001262

6. Greil J, Rausch T, Giese T, Bandapalli OR, Daniel V, Bekeredjian-Ding I, Stutz AM, Drees C, Roth S, Ruland J, et al. Whole-exome sequencing links caspase recruitment domain 11 (CARD11) inactivation to severe combined immunodeficiency. *J Allergy Clin Immunol* (2013) **131**:1376–83 e3.

7. Stepensky P, Keller B, Buchta M, Kienzler AK, Elpeleg O, Somech R, Cohen S, Shachar I, Miosge LA, Schlesier M, et al. Deficiency of caspase recruitment domain family, member 11 (CARD11), causes profound combined immunodeficiency in human subjects. *J Allergy Clin Immunol* (2013) **131**:477–85 e1.
